# Prevalence of virological failure and associated factors among adult individuals on highly active antiretroviral treatment (HAART) in public health facilities at Tulu Bolo Town, Southwest Shoa, Ethiopia, 2024

**DOI:** 10.3389/fpubh.2025.1484866

**Published:** 2025-07-16

**Authors:** Ashenafi Lamesa, Shibire Teshoma, Habtamu Oljira Desta, Belay Tafa Regassa, Tolossa Waqkene

**Affiliations:** ^1^Department of Public Health, Tulu Bolo General Hospital, Tulu Bolo, Ethiopia; ^2^Department of Public Health, College of Medicine and Health Sciences, Ambo University, Ambo, Ethiopia; ^3^Department of Medical Laboratory Sciences, College of Medicine and Health Sciences, Ambo University, Ambo, Ethiopia; ^4^Department of Public Health, Dawo District Health Office, Woliso, Ethiopia

**Keywords:** first line anti-retro viral therapy, virological failure, Tulu Bolo, virologic suppression, HIV positive

## Abstract

**Introduction:**

The goal of antiretroviral therapy for HIV infection is to achieve and maintain virological suppression. Review of charts of adult HIV-positive patients at public health facilities in Tulu Bolo Town reveals that approximately 6.07% of patients did not respond to first-line antiretroviral medication. The identified gap indicates that the study area’s virological failure prevalence and contributing factors are not well-documented. Thus, this study’s objectives are to evaluate prevalence and close a knowledge gap about factors associated with virological failure.

**Objective:**

The objective of this study was to assess the prevalence of virological failure and associated factors among patients taking highly active antiretroviral therapy in Tulu Bolo Town Health facilities, Oromia, Ethiopia, 2024.

**Methods:**

Health facility-based cross-sectional study was conducted at Tulu Bolo Town in public health facilities from 30 September 2024 to 30 October 2024. The total sample sizes of 274 records of clients were selected using simple random sampling technique. Data were cleaned and entered into EPI info version 7.2.0.1 and exported to SPSS version 20.0 for further analysis. The association was identified using binary logistic regression model. An adjusted odds ratio with 95% confidence intervals (CI) was computed to identify the presence and strength of association. Finally, statistically significant variables were declared at a *p*-value of < 0.05 along with 95% CI.

**Results:**

A total of 274 charts of HIV-positive clients were included in the study. The magnitude of virological failure was 12.8% (95%CI 9, 17%). Baseline CD4 count < 200 (AOR 6.1, 95%CI 2.06, 18.43), clients infected with TB (AOR 4.8 95%CI 1.78, 12.96), treatment interruption (AOR 3.05, 95% CI 1.06, 8.77), and adherence (AOR 3.67, 95%CI 1.39, 9.66) were statistically significant association.

**Conclusion and recommendation:**

The overall prevalence of virological failure of this study was high as compared to standard. Baseline CD4 count, TB infection, treatment interruption, and adherence were significant factors. Health facility ART provider and HIV/AIDS program manager should give special attention for clients with history of TB co-infection and CD4 count < 200 needs care and support and providing TB preventive therapy.

## 1 Introduction

Human immunodeficiency virus (HIV) is one of the most common chronic health conditions that attack the immune system, and the risk of HIV transmission is proportional to HIV viral load. Sub-Saharan Africa bears the greatest burden of the worldwide HIV epidemic, with over 75% of fatalities and 65% of new infections in 2017. The majority of persons living with HIV live in low- and middle-income nations (1).

Assessment of virological outcomes in the adult population receiving ART in Ethiopia remains a public health problem. According to a study conducted in Ethiopia from March 2016 to 2017, the rate of virological failure among Ethiopians taking ART is 11% (2). Drug resistance and treatment failure are key barriers for successful viral suppression in resource-constrained nations like Ethiopia, affecting the effective aims of first-line HAART (3).

Patients who are unable to work (first non-working functional status, i.e., ambulatory/bed ridden) due to a health problem are 3.5 times more likely to have virological failure (4). Similarly in Tulu Bolo Town public health facilities, approximately 6.07% of adult individuals on HAART are switched to second-line drug regimen due to failure of first-line ART treatment, and in addition to this, the contributing factors for virological failure are increasing from time to time.

To halt this, effective antiretroviral therapy (ART) inhibits viral replication and reduces HIV viral load to low or undetectable levels. Early ART initiation significantly reduces the risk of HIV transmission (5).

The World Health Organization (WHO) suggests using clinical and immunological criteria to track treatment failure in resource-constrained situations. These factors have been proved to be poor markers of treatment failure, resulting in missed opportunities or unnecessary medication shifts, which not only increases treatment expenses but also limits future therapy possibilities (6).

Treatment success for HIV/AIDS patients on combination antiretroviral medications is defined as a viral load <1,000 copies/ml. According to studies, undetectable blood plasma viral load requires at least 6 months of regular ART. Although viral load is the recommended method for identification and confirmation of treatment failure, research into its prevalence and determinants is limited (7).

If viral load is not routinely available, the CD4 count and clinical monitoring should be utilized to determine therapy failure (8). Monitoring HIV viral load suppression status in people living with HIV is the core to sustain effective individual ART and monitoring progress toward meeting global 95% targets for viral suppression (9).

Most HIV programs generally underestimate the frequency of treatment interruptions, and HIV care is more challenging for individuals previously exposed to ART and at risk of HIV drug resistance, especially if presenting back to care with advanced HIV disease (10).

Patients whose VLs are not suppressed at retesting can be classified as having virological failure and an elevated or non-suppressed viral load (>1,000 copies/ml) in a patient who has been on ART for at least 6 months can indicate either therapeutic failure due to antiretroviral resistance and/or poor adherence to treatment. To distinguish between these two conditions, a patient with an elevated VL should receive adherence support followed by retesting 3–6 months later (11).

The WHO immunological criteria for monitoring of response to ART have low sensitivity and positive predictive value in detecting treatment failure. As a result, depending on CD4 counts for treatment, monitoring could lead to incorrect diagnoses of treatment failure, resulting in an inappropriate or delayed transfer to second-line ART. Viral load monitoring is critical to avoiding these misclassifications, the challenge of transitioning to second-line ART, and the possibility of medication resistance. The most accurate and significant metric of ART success is monitoring the amount of HIV RNA to demonstrate optimal treatment response and long-term viral suppression (12).

Antiretroviral treatment failure can be prevented by implementing globally recommended strategies such as improving ART adherence, taking medication based on the appropriate prescription, increasing patients’ knowledge and attitudes toward HAART, timely ART initiation, prevention and control of opportunistic infections, and the implementation of effective food and nutrition policies (3).

Antiretroviral therapy (ART) for HIV infection aims to achieve and maintain virological suppression, thereby slowing disease development and transmission (13). Antiretroviral medication is recommended for all HIV patients to help them live longer, healthier lives and reduce the risk of HIV transmission. Ethiopia has endorsed the WHO guideline to provide lifelong ART to all people living with HIV, including children, adolescents, and adults, and pregnant and breastfeeding women, regardless of clinical status or CD4 cell level (14).

A VL of more than 1,000 copies/ml in a patient who has been on ART for at least 6 months may indicate therapeutic failure due to antiviral resistance or poor adherence. Virological failure is associated with long-term therapy, age ≥ 35 years at ART start, TB diagnosis during ART, poor adherence, low NNRTI plasma concentration, and non-specific symptoms (e.g., myalgia, hepatomegaly, splenomegaly, and lymphadenopathy) (15).

According to the Ethiopian viral load testing hypothesis, there are three main reasons for viral load testing: (1) routine testing; (2) suspected failure testing; and (3) repeat viral load testing after suspected treatment failure for patients who were virologically non-suppressed on first time testing and underwent enhanced adherence support for 3 months and then repeat 3–6 months before switching to second-line drug (16).

## 2 Methods

### 2.1 Study area and period

The study was carried out in Tulu Bolo Town, which is located in Southwest Shoa Zone, Oromia regional state, 80 kilometers from Addis Ababa on the Addis Ababa-Jimma Road. The town has a total of 20,058 people. The town contains one Health Center and one General Hospital, both of which offer ART services. In all, 675 persons living with HIV were receiving ART at the town’s two public health facilities. Data were taken from 30 September to 30 October 2024.

### 2.2 Study design

Health facility-based cross-sectional study design was used to conduct the study.

### 2.3 Population

#### 2.3.1 Source population

Source population includes all adult ART clients and those on follow-up of ART attending Tulu Bolo General Hospital and Tulu Bolo Health Center ART clinic.

#### 2.3.2 Study population

The study papulation includes all records of adult (≥18 years) human immunodeficiency virus clients and greater than or equal to 6 months on HAART during study period in Tulu Bolo General Hospital and Tulu Bolo Health Center ART clinic.

#### 2.3.3 Inclusion and exclusion criteria

##### 2.3.3.1 Inclusion criteria

The inclusion criteria were all records of adult patients on ART for greater than or equal to 6 months with recorded viral load result during the study period.

##### 2.3.3.2 Exclusion criteria

The exclusion criteria were incomplete records of clients.

### 2.4 Sample size determination and sampling technique

#### 2.4.1 Sample size determination

The sample size was estimated using the single population proportion calculation, taking into account the assumption of a 95% level of confidence, a 5% margin of error, and the proportion of viral failure (20.3%) from a prior study done in Southwest Ethiopia (4) (Table 1).


$$n=\frac{d}{2}$$

$$n=\frac{{\left(1.96\right)}^{2}(0.203)(0.797)}{{\left(0.05\right)}^{2}}$$

**Table 1:** Objective 2 sample size determination for factors associated with virological failure among adults on HAART at Tulu Bolo Town in public health facilities, Southwest Shoa, Oromia, Ethiopia, 2024.

Main factors	Confidence interval	Power	Ratio	Proportion of controlled exposed (%)	AOR	Sample size	Non-response rate (10%)	Total sample size	References
CD4 count	95%	80	1:1	23.6	3.0	158	16	174	Abebaw (16)
TB confections	95%	80	1:1	23.2	3.7	170	17	187	Abebaw (16)
Discontinuation of ART	95%	80	1:1	23.1	3.5	105	11	116	Abebaw (16)

After comparing the estimated sample size for objective 1 and objective 2, the largest sample size was selected as the final sample sizes, which were 274.

*n* = 249.

Considering 10% contingency (e.g., incomplete data), the final sample size was = 274.

Where

P is the proportion of virological failure (20.3%) from previous study which was conducted in Southwest Ethiopia (4), Z α/_2_ is the value corresponding to confidence level of (95%), ɖ is the margin of error between the sample and population (0.05), and *n* is the estimated sample size.

### 2.5 Sampling technique

**Figure 1:**
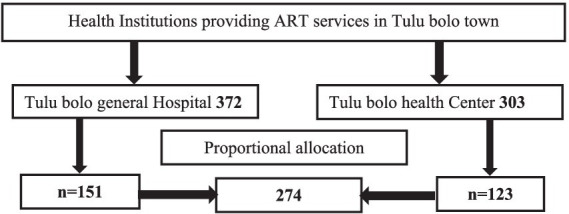
Sampling frame for determining virological failure and associated factors of adult HIV-positive patients in Tulu Bolo Town public health facilities, Oromia, Ethiopia, 2024.

Charts for adult ART clients were selected from the records using a simple random sample method. The sample frame was created using the unique identifying numbers from each health facilities based on eligibility requirements. The appropriate sample size was determined from the prepared sample frame using computer-generated random numbers, and the total sample size was assigned proportionally to each health facility (Figure 1). Data were taken from 274 patient records.

### 2.6 Study variables

#### 2.6.1 Dependent variable

Virological failure is considered as a dependent variable.

#### 2.6.2 Independent variables

The independent variables are as follows:

**Socio-demographic-related variables:** Age, sex, marital status, occupational status, and educational status, residence area.

**Clinical and laboratory-related variables**: WHO clinical stages, baseline CD4 count, history of opportunistic infection, TB co-infection, functional status, nutritional status.

**Medication-related variables**: Baseline ART regimen, duration on ART, treatment interruption, disclosure status, current regimen, and substance use and drug adherence level.

### 2.7 Operational definitions

#### 2.7.1 Virological failure

It refers to viral load above 1,000 copies/mL based on two consecutive viral load measurements 3 months apart, with adherence support following the first viral load test, by using Quantitative Real-Time PCR HIV-1 assay by the COBAS^®^ Amplify Prep instrument (Roch, Homburg, Germany) Plasma (17, 18).

#### 2.7.2 Antiretroviral therapy

It refers to the use of anti-HIV medications to treat HIV infections. HIV replication is suppressed by a combination of medications known as “highly active antiretroviral therapy” (HAART). This combination of medications has the potential to boost potency and diminish viral resistance (1).

#### 2.7.3 Viral load non-suppression

It refers to elevated viral load in RNA copies of ≥1,000 per ml in plasma of patients who has been on ART for at least 6 months (9).

#### 2.7.4 Adherence

It is defined as good adherence when >95% and poor adherence when <85% doses taken (19).

#### 2.7.5 Good adherence

Good adherence is defined as when the average treatment adherence of HIV patients is greater than or equal l–95% (2).

#### 2.7.6 Poor adherence

If a client used less than 85% adherence, that is, missing ≥5 doses out of 30 doses or more than 10 doses from 60 doses, then it is referred to as poor adherence (20).

### 2.8 Data collection tool

To get the relevant data from record review, a data extraction form or information collecting sheet was constructed utilizing the FMOH of Ethiopia’s ART follow-up form and patient cards. The checklist includes questions about socio-demographic parameters, clinical and laboratory-related factors, other health-related diseases, and medication-related variables.

### 2.9 Data collection procedure

Data were acquired by studying the patient’s medical record. The data were collected by one BSc nurse and one data clerk from the ART clinic at Tulu Bolo Hospital, as well as one BSc nurse and one data clerk from the ART clinic at Tulu Bolo Health Centre.

### 2.10 Data quality control and management

Data were gathered from the patient’s medical record, which was reviewed for completeness and inconsistency at the time of collection. The primary investigator trained data collectors for 1 day on the study’s objectives, how to complete the data extraction form, and how to handle the information they obtained. There was also rigorous supervision during the data collection process. The principal investigator and supervisor ensured that the data were properly categorized and coded, as well as its accuracy and clarity.

### 2.11 Data processing and analysis

Data were cleaned and entered to EPI info version 7.2.0.1 and exported to SPSS version 20.0 for further analysis. The data were presented using descriptive statistics such as frequencies, proportions, tables, and texts. Bivariable logistic regression and multivariable logistic regression analyses were used to identify the presence of associations. All independent variables that were significant at a *p*-value of < 0.25 in bivariable analysis were considered for multivariable logistic regression analysis for controlling the possible effect of confounding variables. To investigate the strength of association between dependent and independent variables, adjusted odds ratio was computed at 95% confidence interval and variables with a *p*-value of < 0.05 in regression analysis were declared for statistical significant association. The fitness of the model was tested by Hosmer–Lemeshow goodness-of-fit test, multi-collinearity was checked using variance inflation factor (VIF), and the result of VIF calculation was < 10.

## 3 Results

### 3.1 Socio-demographic characteristics of study population

The study comprised 274 HIV-positive adult clients’ records. The response rate was one 100%. Of the 274 ART charts, 106 (38.7%) were aged ≥45 years. During first-line ART, customers’ median age was 42 years (IQR 35–50). More than half of the clients, 174 (63.5%), were men. In terms of marital status, 146 (53.3%) of the respondents were married. In terms of educational standing, 180 (65.7%) of the participants received formal education, while 147 (53.6%) of the clients lived in rural areas. The majority of the client, 131 (47.8%), were farmers (Table 2).

**Table 2:** Socio-demographic characteristics of adult HIV-positive patients in Tulu Bolo public health facilities, Oromia, Central Ethiopia, 2024.

Variables	Frequency	Percent
Age of clients during first-line ART		
15–24	14	5.1%
25–34	55	20.07%
35–44	99	36.13%
≥45	106	38.7%
Sex of clients		
Male	174	63.5%
Female	100	36.5%
Educational status		
No formal education	94	34.3%
Formal education	180	65.7%
Marital status		
Single	35	12.8%
Married	146	53.3%
Widowed and separated	74	27%
Divorced	19	6.9%
Residence of clients		
Rural	147	53.6%
Urban	127	46.4%
Occupational status		
Farmer	131	47.8%
Government employee	44	16.1%
House wife	54	19.7%
Non-government employee	45	16.4%

### 3.2 Clinical and laboratory-related characteristics of virological failure among HIV-positive adult patients

The study’s overall virological failure rate was 12.8% (95% confidence interval [CI] 9 to 17%). In terms of nutritional status, the majority of clients, 177 (64.6%), had normal nutritional status, which was defined as having a body mass index greater than or equal to 18.5 kg/m2, whereas 97 (35.4%) of clients were undernourished, meaning their BMI was less than 18.5 kg/m^2^.

Approximately 33 (12%) of patients were in WHO clinical stage III or IV when they began HAART treatment. In terms of baseline functional state, approximately 251 (91.3%) were working, 16 (5.8%) were ambulatory, and 7 (2.5%) were bedridden when ART was initiated. Approximately 50 (18.2%) of clients had a history of opportunistic infections. The majority of consumers had a baseline CD4 cell count of more than 200 cells per microliter of blood (182; 66.4%). Of the total clients, approximately 73 (26.6) had a history of tuberculosis infection (Table 3).

**Table 3:** Clinical and laboratory measures of virological failure among adult ART patients in Tulu Bolo Town health facilities, Oromia, Central Ethiopia, 2024.

Variables	Variable category	Frequency	Percent
Level of virological status	Failed	239	87.2%
	Not failed	35	12.8%
WHO clinical stage	I and II	241	88%
	III and IV	33	12%
Baseline CD4 measurement	<200	92	33.6%
	≥200	182	66.4%
Opportunistic infection	Yes	50	18.2%
	No	224	81.8%
TB co-infection	Yes	73	26.6%
	No	201	73.4%
Baseline functional status	Working	251	91.3%
	Ambulatory	16	5.8%
	Bedridden	7	2.5%
Nutritional status	Under nutrition	97	35.4%
	Normal	177	64.6.%

### 3.3 Medication-related characteristics of virological failure among HIV-positive adult patients in Tulu Bolo Town, 2024

Regarding initial ART regimen, majority of clients, 161 (58.8%), were on TDF + 3TC + EFV followed by TDF + 3TC + DTG [58 (21.2%)], ZDV + 3TC + NVP [47 (17.1%)], and D4T + 3TC + NVP [8 (2.9%)], respectively. The majority, 228 (83.2%), of HIV-positive clients had disclosed their HIV sero status to their family. The median duration of clients on first-line ART was 98 months. Most of the clients, 248 (90.6%), currently on first-line ART used DTG0based TDF + 3TC + DTG regimen. Concerning duration on ART, more than three-forth, 237 (86.5%), of the clients have taken the drug for greater than or equal to 36 months. Approximately 199 (72.6%) of HIV-positive ART clients had good adherence to drug, and 75 (27.4%) of clients had poor drug adherence. Regarding to substance use, the majority, 232 (84.7%), not used any substance, and some of them, approximately 42 (15.2%), used certain substance. Regarding treatment interruption, approximately 82 (29.9%) adult ART clients had history of treatment interruption, and most, 192 (70.1%), of them had not interrupted their treatment (Table 4).

**Table 4:** Medication-related factors of virological failure among adult ART patients in Tulu Bolo Town health facilities, Oromia, Central Ethiopia, 2024.

Variables	Variable category	Frequency	Percent
Adherence to drug	Poor	75	27.4%
	Good	199	72.6%
Treatment interruption	Yes	82	29.9%
	No	192	70.1%
Duration on ART	<36 months	37	13.5%
	≥36 months	237	86.5%
Original first-line ART	TDF + 3TC + EFV	161	58.8%
	ZDV + 3TC + NVP	47	17.1%
	TDF + 3TC + DTG	58	21.2%
	D4T + 3TC + NVP	8	2.9%
Current treatment category	DTG based (TDF + 3TC + DTG)	248	90.2%
	EFV based (TDF + 3TC + EFV)	23	8.4%
	Others first-line ART drug	3	1.4%
Disclosure status	Disclosed	228	83.2%
	Not disclosed	46	16.8%
Substance use	Used	42	15.3%
	Not used	232	84.7%

### 3.4 Bivariable analysis results of independent variables of virological failure among adult HIV-positive patients

In bivariable analysis, there were some independent variables associated with the outcome variable. Among them, age of clients during first-line ART 25–34 (COR 0.14, 95%CI 0.03, 0.65, *p*-value 0.01), clients age 35–44 (COR 0.18, 95%CI 0.05, 0.65, *p*-value 0.09), sex of client (COR 2.02, 95%CI 0.99, 4.14, *p*-value 0.05), marital status (COR 3.72, 95%CI 0.41, 33.52, *p*-value 0.26), baseline CD4 measures <200 (COR 16.33, 95% CI 6.37, 46.09, *p*-value 0.001), treatment interruption (COR 13.7, 95% CI 5.67, 33.1, *p*-value 0.001), and TB co-infection at initiation of ART (COR 14.16, 95%CI 6.04, 33.19, *p*-value 0.001) were some of the associated variables with virological failure in bivariate analysis (Table 5).

**Table 5:** Bivariate analysis for association of outcome and independent variables in Tulu Bolo Town public health facilities, Oromia, Central Ethiopia, 2024.

Variables		Virological failure				
	Category	Yes	No	COR	95% CI	*p*-value
Age of clients during first-line ART	15–24	5 (1.8%)	9 (3.2%)	1		
	25–34	6 (2.1%)	49 (17.9%)	0.14	(0.03, 0.65)	0.01
	35–44	9 (3.2%)	90 (32.8%)	0.18	(0.05, 0.65)	0.09
	≥45	15 (5.4%)	91 (33.2%)	0.33	(0.10, 1.12)	0.07
Sex	Female	18 (6.6%)	82 (29.92%)	2.02	(0.99, 4.14)	0.05
	Male	17 (6.2%)	157 (57.29%)	1		
Marital status	Single	6 (2.1%)	26 (9.5%)	1		
	Married	13 (4.74%)	129 (47.08%)	0.63	0.23, 1.75	0.38
	Widowed and separated	11 (4.01%)	63 (23.0%)	0.84	0.28, 2.50	0.76
	Divorced	5 (1.8%)	18 (6.6%)	0.26	0.03, 2.41	0.26
Nutritional status	Under nutrition	15 (5.5%)	82 (29.92%)	1.43	(0.67, 2.95)	0.32
	Normal	20 (7.3%)	157 (57.3%)	1		
Baseline CD4 measurement	<200	30 (10.9%)	62 (22.6%)	16.33	(6.33, 46.09)	0.001
	≥200	5 (1.8%)	177 (64.6%)	1		
Treatment interruption	Yes	28 (10.2%)	54 (19.7%)	13.7	(5.67, 33.1)	0.001
	No	7 (2.5%)	185 (67.51%)	1		
TB co-infection	Yes	27 (9.9%)	46 (16.8%)	14.16	(6.04, 33.19)	0.001
	No	8 (2.9%)	201 (73.4%)	1		
Duration on ART	<36 months	5 (1.8%)	32 (11.7%)	1.07	(0.39, 2.98)	0.88
	≥36 months	30 (10.9%)	207 (75.5%)	1		
HIV disclosure status	Disclosed	27 (9.9%)	201 (73.4%)	1		
	Not disclosed	38 (13.9%)	8 (2.9%)	1.56	(0.66, 3.71)	0.30
Level of adherence	Poor	26 (9.5%)	49 (17.9%)	11.2	(4.93, 25.44)	0.001
	Good	9 (3.3%)	90 (69.34%)	1		
Substance used	Substance used	6 (2.2%)	36 (13.13%)	1.16	(0.45, 3.01)	0.75
	Substance not used	29 (10.6%)	203 (74%)	1		
WHO clinical stage	Stages I and II	6 (2.2%)	29 (10.6%)	1		
	Stages III and IV	27 (9.9%)	212 (77.4%)	1.62	(0.61, 4.26)	0.32
Opportunistic infection	Yes	8 (2.9%)	42 (15.3%)	1.38	(0.59, 3.27)	0.45
	No	27 (9.6%)	197 (71.2%)			

### 3.5 Multivariable analysis of independent variables of virological failure among adult HIV-positive patients

The odds of virological failure among clients with baseline CD4 count <200 cells/microliter were more than six times higher to occur as compared to those with baseline CD4 cell count ≥200 cells/microliter (AOR 6.1, 95%CI 2.06, 18.43). The odds of virological failure among clients infected with TB were more than 4-fold as compared to those with no history of TB confection (AOR 4.8, 95% CI 1.78, 12.96). The odds of virological failure among HIV-positive clients with history of treatment interruption were approximately three times as compared to those who have not interrupted their treatment (AOR 3.05, 95% CI 1.06, 8.77). The odds of virological failure among clients with poor drug adherence were more than 3.67-fold as compared to clients with good drug adherence (AOR 3.67, 95%CI 1.39, 9.66) which were independent variables that have statistically significant association with virological failure (Table 6).

**Table 6:** Independent predictors of virological failure in Tulu Bolo Town public health facilities, Oromia, Ethiopia, 2024.

		Virological failure				
Variables	Category	Yes	No	AOR	95% CI	*p*-value
Baseline CD4 count	<200	30 (10.9%)	62 (22.6%)	6.19	(2.06, 18.43)	0.001**
	≥200	5 (1.8%)	177 (64.6%)	1		
Treatment interruption	Yes	28 (10.2%)	54 (19.7%)	3.05	(1.06, 8.77)	0.038**
	No	7 (2.5%)	185 (67.51%)	1		
TB co-infection	Yes	27 (9.9%)	46 (16.8%)	4.8	(1.78, 12.96)	0.002**
	No	8 (2.9%)	201 (73.4%)	1		
Adherence level	Poor	26 (9.5%)	49 (17.9%)	3.67	(1.39, 9.66)	0.008**
	Good	9 (3.3%)	190 (69.34%)	1		
Sex of clients	Female	18 (6.6%)	82 (29.92%)	1.87	(0.73, 4.76)	0.18
	Male	17 (6.2%)	157 (57.29%)	1		
Clients age during first-line ART	15–24	5 (1.8%)	9 (3.2%)	1		
	25–34	6 (2.1%)	49 (17.9%)	0.26	(0.04, 1.68)	0.15
	35–44	9 (3.2%)	90 (32.8%)	0.43	(0.04, 1.03)	0.06
	≥45	15 (5.4%)	91 (33.2%)	0.28	(0.06, 1.35)	0.11

**Significant at *p*-value < 0.05 (adjusted odds ratio).

## 4 Discussion

Continuous virological failure is related to difficulty to delivering quality of care, the emergence of drug resistant viruses which limits the treatment option and increases the threat of morbidity and mortality. So this study was intended to assess the prevalence and associated factors of virological failure among adult HIV/AIDS clients.

In this study, the virological failure was 12.8%. The finding of this study was almost similar with studies carried out in study in India which indicates that the proportion of virological failure among adult HIV-positive patients who were on first-line antiretroviral therapy was 13% (21). The prevalence of this study was almost similar with study conducted in Tigray regional state at Adigrat general hospital, which reveals that the overall virological failure of adult HIV-positive clients on highly active antiretroviral therapy was 12.47% (2). The prevalence of virological failure of this study was also consistent with study done in University of Gondar which indicates that the magnitude of virological failure was 14.7% (19).

On the other hand, the prevalence of virological failure of this study was lower than the prevalence of virological failure of study conducted in Lesotho which reveals that Lesotho Population-based Health Impact Assessment results showed prevalence of 32.2% (22). This is probable due to difference in sample size in which sample size of this study was lower than that of study conducted in Lesotho and also the scope of study conducted in Lesotho includes approximately eight clinics and one hospital which covered more areas than this study.

This study identified that the odds of virological failure among clients with baseline CD4 count <200 cells/microliter were more than six times higher as compared to those with baseline CD4 cell count ≥200 cells/microliter. Similar study conducted in Debre Markos, North East Ethiopia, showed that the odds of virological failure among clients with baseline CD4 count < 200cells/microliter were three times higher when compared to HIV-positive clients with CD4 cells count ≥ 200cells/microliter in which the probability of developing virological failure of patients with CD4 count <200 cells/microliter is similar to this study (16).

Another study conducted in Sekota, North East Ethiopia, indicates that the odds of virological failure among clients with CD4 cell count <200 cells/microliter were nearly five times to occur as compared to those who had CD4 cell count ≥200cells/microliter (18), which was almost similar to this study which indicates that the odds of virological failure among clients with baseline CD4 count <200 cells/microliter were more than six times higher as compared to those with base line CD4 cell count ≥200 cells/microliter. It is evident that a higher degree of viral replication is common among patients with a decreased level of CD4 count and subsequently leads to virological failure.

Study conducted in Uganda shows that CD4 count at ART initiation is a predictor of virological suppression. According to this study, virological failure of ≥ 6 months after the start of ART was associated with lower subsequent CD4 cell counts, with greater CD4 cell count reduction for more recent virological failure and higher viral load (23). This was also similar with this study in which lower CD4 count was a predictor of virological failure.

Treatment interruption was strongly associated in this study. The odds of virological failure among HIV-positive clients with history of treatment interruption were approximately three times as compared to those who have not interrupted their treatment. This study is supported by study done in Debre Markos, North East Ethiopia, which indicates that discontinuation or interruption of treatment was found to be statistically significant determinant factors of virological failure. It shows that the odds of virological failure of HIV-positive clients who had interrupted ART to take other traditional herbal medicine and holy water were three times as compared to those clients who had not interrupted their treatment ART (16).

On another hand, this study is inconsistent with the study conducted in the University of Gondar Hospital, North West Ethiopia, in which history of treatment interruption is not independently associated with virological failure (16, 19).

The possible reason for discrepancy may be, due to sample size used, study design used and the sources of data used in Gondar University were both primary and secondary which is more accurate than data source of this study in which only secondary data used and the strategies used for ART client management may be different.

According to this study, history of TB infection is another independent risk factor of virological failure. This study indicates the odds of virological failure among clients infected with TB were more than 4-fold higher as compared to those with no history of TB confection. The finding of this study was strongly supported with study that has been done in Adigrat general hospital, Northern Ethiopia (2).

This study reveals that the odds of virological failure among clients on antiretroviral treatment with poor drug adherence were more than 3.67-fold higher as compared to clients with good drug adherence. This study was comparable with the study done in Amhara Regional State, North East Ethiopia, which indicates that the odds of virological failure among HIV-positive clients with poor drug adherence were approximately 10-fold as compared to HIV clients with good drug adherences (24).

## 5 Limitation of the study

This study used data from patient medical records (secondary data), which may have excluded additional key contributing variables for virological failure due to the nature of secondary data. The study is based solely on secondary data from the past, which can alter from time to time.

## 6 Conclusion

This study revealed a large amount of virological failure in the study setting. Our findings underscore the need for increased commitment and effort from all stakeholders on these factors to ensure HAART’s long-term success. The findings demonstrate that virological failure is a problem in a situation where highly active antiretroviral medication has been widely implemented. The issue is particularly prevalent in people who do not stick to their medication regimen. This will have an impact on global plans for 90% viral suppression. This could imply a need for increased investment and commitment to increasing patient adherence in the research area. Adult ART patients who experienced treatment interruption, had a baseline CD4 cell count <200 cells/microliter, had a history of tuberculosis infection at the time of ART initiation, and had low adherence were more likely to experience virological failure. Adherence, co-infection, advanced WHO clinical stage, regimen modification, and disclosure all contribute to treatment failure. As a result, enhanced treatment adherence, co-infection avoidance, and prompt and rigorous follow-up were found to maintain first-line therapeutic efficacy and prevent HIV drug resistance. Therefore, due attention should be given to patients with these identified factors.

In conclusion, the findings of this study are practical and crucial for achieving viral suppression among HIV/AIDS patients in Ethiopia, and they contribute to the global literature on HIV/AIDS care. The following points should also be addressed.

- Healthcare providers should regularly follow the patients to avoid treatment interruption.
- Since reliance on CD4 and clinical criteria may result in delayed diagnosis of treatment failure, great concern should be given for ART clients with history of TB infection as well as care and treatment adherence counseling and giving TB preventive therapy for eligible clients.
- Baseline viral load test for every patient should be done which alerts healthcare providers to suspect risk of treatment failure.
- HIV-positive clients should understand the risk of virological failure and adhere to treatments with regular follow-up.
- Program manager and local planners should strengthen monitoring of virological failure and early identification of associated factors to benefit patients to prevent from further complication.
- Clients with baseline CD4 < 200 cells/microliter require additional support to correctly adhere to treatment to boost their immune system.
- Using the finding of this study as baseline data, other researchers should conduct further studies and address factors associated with virological failure which was not discussed under this study.
- Further assessment of the effectiveness of antiretroviral drug is needed to control the HIV pandemic.

## Data Availability

The original contributions presented in the study are included in the article/supplementary material, further inquiries can be directed to the corresponding author.
